# AlphaLISA detection of alpha-synuclein in the cerebrospinal fluid and its potential application in Parkinson’s disease diagnosis

**DOI:** 10.1007/s13238-017-0424-4

**Published:** 2017-05-29

**Authors:** Hongli Zhao, Jue Zhao, Jiapeng Hou, Siqing Wang, Yu Ding, Boxun Lu, Jian Wang

**Affiliations:** 1 State Key Laboratory of Medical Neurobiology, Department of Neurology in Huashan Hospital, School of Life Sciences, National Clinical Research Center for Aging and Medicine, Fudan University, Shanghai, 200040 China; 2 Changzhou Furuite Biological Technology Co. Ltd., Changzhou, 213145 China; 3 Collaborative Innovation Center for Brain Science, Fudan University, Shanghai, 200032 China

**Dear Editor,**

Parkinson’s disease (PD) is the second most prevalent neurodegenerative disorder affecting about 1% of the worldwide population over the age of 60 (El-Agnaf, 2003; Majbour, Vaikath et al., 2016). Motor symptoms, which is currently the major trait for PD diagnosis, appear when 50%–60% of dopaminergic neurons in the substantia nigra (SN) and 70%–80% of dopaminergic terminals in the striatum are lost (Kim, Paik et al., 2014; Landeck, Hall et al., 2016), making PD extremely challenging to treat when diagnosed. Thus, PD biomarkers other than the motor symptoms may provide additional help in PD diagnosis, making earlier PD treatment possible to help the management and treatment of the disease. One of the pathological hallmarks of PD is the presence of Lewy bodies (LBs) and Lewy neurites in surviving PD neurons (Delenclos, Jones et al., 2016; Majbour, Vaikath et al., 2016). LBs contain a large amount of accumulated alpha-synuclein proteins (α-syn), which is a 14.6 kDa lipid-binding protein and encoded by the *SNCA* gene (De Genst, Guilliams et al., 2010, Bartels, Choi et al., 2011). The accumulation and aggregation of α-syn is likely contributing to PD progression. The α-syn variant A53T (53 A→T) found in human patients has no effect on osmotic stress-induced phosphorylation, but increases oligomerization and exacerbates disease progression; suggesting a causal relationship between α-syn oligomerization and PD (Berrocal, Vasquez et al., 2014). Consistently, recent work suggested that monomeric α-syn level is likely reduced in cerebrospinal fluid (CSF) of the PD patients, while the oligomeric α-syn may increase (Aasly, Johansen et al., 2014). Meanwhile, the results have been controversial possibly due to lack of sensitive and reliable detection methodologies for CSF α-syn detection, especially for the α-syn oligomers.

Here we established a robust and reproducible AlphaLISA assay that allows sensitive and reliable measurements of the relative levels of oligomeric versus total α-syn directly in the microliter wells. AlphaLISA signals are generated only when the acceptor-bead conjugated antibody and the donor-bead conjugated biotinylated antibody bind to the same target protein molecule, which brings the acceptor and donor beads to close proximity (<200 nm) so that the single O_2_ molecules could be transferred from the donor to the acceptor (Bielefeld-Sevigny, 2009). We utilized the Life/Nb antibody pair (see supplement for antibody information) to detect the total α-syn. These antibodies bind with different α-syn epitopes, and thus both the monomeric and the oligomeric α-syn can bind with the two antibodies simultaneously, generating AlphaLISA signals (Fig. S1A). For α-syn oligomer detection, we utilized the Life/Life antibody pair. In this case, the same antibody (Life) was conjugated with both the acceptor and the donor beads. A single monomeric α-syn molecule has only one binding epitope for the Life antibody and thus could not bind with both the acceptor- and the streptavidin donor- conjugated Life antibodies at the same time, and thus cannot generate any AlphaLISA signals (Fig. S1B). Meanwhile, oligomeric α-syn protein molecules can generate AlphaLISA signals by providing multiple epitopes for the Life antibody (Fig. S1B).

To validate the scenario above, we utilized recombinant purified α-syn protein suspension that contains both monomeric and oligomeric forms (Fig. 1A), both the Life/Nb and Life/Life antibody pair generates detectable and linear AlphaLISA signals for this purified protein sample (Fig. 1B and 1C), suggesting that the AlphaLISA signals faithfully reflect the relative α-syn levels. The linear detection range includes at least from 3.25 ng/mL to 200 ng/mL, and the α-syn levels in CSF are likely within this range. We also tested this design with TR-FRET (Fig. S2A and S2B), which was utilized in other studies for α-syn detection (Bidinosti M. 2012). TR-FRET assays detected a largely linear signal for the α-syn protein, but the variation was higher than AlphaLISA signals for every concentration tested, and the linearity was largely abolished at concentrations below 25 ng/mL (Fig. S3C and S3D). Thus, AlphaLISA is a superior assay for α-syn detection, at least using these antibody pairs.

**Figure 1:**
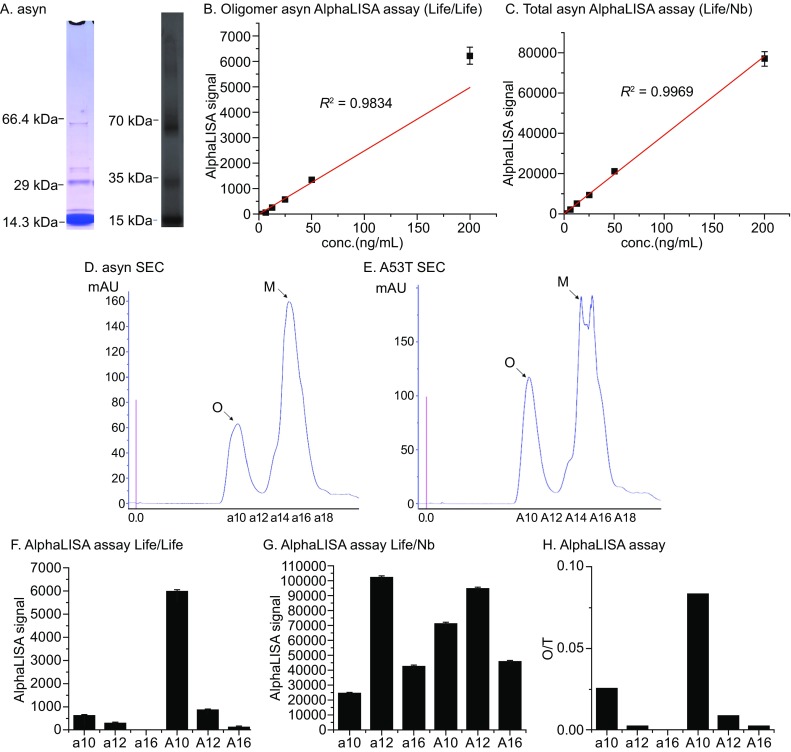
Development of AlphaLISA assays for detection of oligomeric and total α-syn. (A) Coomassie staining (blue) and Western-blot (dark, using Life antibody) analysis of recombinant purified α-syn, which contains both monomeric and oligomeric α-syn. (B) AlphaLISA assay of the purified protein in (A) uses the Life /Life antibody pair. The protein was tested at different concentrations indicated in the X-axis and the signals were fitted with Y = kX. *R* ^2^ indicates the regression parameter. (C) Similar as (B), but using the Life/Nb antibody pair. (D) α-Syn protein was separated from oligomeric α-syn and monomeric by SEC. (E) Similar as (D), but using A53T protein. (F) The Life/Life antibody pair AlphaLISA detection of the indicated α-syn protein samples from SEC at 1 μg/mL on 384-well microtiter plates (*n* = 3). The monomeric samples a12&a16, A12&A16 generated much smaller signals compared to oligomeric samples a10 and A10. (G) Similar as (F), but using the Life/Nb antibody pair. All samples gave signals. (H) The ratio between Life/Life signals and the Life/Nb signals was calculated for the AlphaLISA assay, and this ratio (O/T) separates oligomeric and monomeric samples. For Figure 1B–G, plots indicate mean ± SEM, and *n* = 3

To determine whether the Life/Life antibody pair detects oligomeric α-syn specifically, we purified different forms of wild-type and A53T α-syn proteins by size-exclusion chromatography (SEC) (Fig. 1D and 1E), and validated the separation by electrophoresis under native conditions (Fig. S2C and S2D). The a10 fraction mainly contains oligomeric α-syn at high molecular weight (Fig. S2C, lane 2) whereas the a12 and a16 fraction mainly contain monomer (Fig. S2C, lanes 3 and 4). Similarly, A10 fraction mainly contain oligomeric A53T α-syn (Figs. 1E and S2D, lane 2), whereas A12 and A16 were mainly monomer (Fig. S2D, lanes 3 and 4). The native PAGE gel takes less charge so that the apparent molecular weight could be deviated from the predicted molecular weight.

We then tested different α-syn forms from the above SEC samples with both AlphaLISA and TR-FRET assays. Consistent with our design, in which the Life/Nb antibody pair detects total α-syn, AlphaLISA signals from all the fractions tested were detected using this antibody pair (Fig. 1G for AlphaLISA and Fig. S2F for TR-FRET). In comparison, when using the Life/Life antibody pair, only the oligomeric α-syn containing samples including a10, and A10 gave detectable signals while the monomeric fractions a16, A16 generated essentially no signal (Fig. 1F for AlphaLISA and Fig. S2E for TR-FRET), confirming that the Life/Life antibody pair specifically detects oligomeric α-syn. To further exclude the influence of the protein loading, we calculated the ratio between Life/Life versus Life/Nb signals (O/T), as an indicator of oligomerization. It is clear that a10 and A10 fractions have the highest O/T in AlphaLISA assays (Fig. 1H for AlphaLISA and Fig. S2G for TR-FRET), consistent with our SEC and native electrophoresis results showing that they have highest oligomerization. The data above confirm that the Life/Life antibody pair detects oligomeric α-syn, and the ratio between the Life/Life signal and the Life/Nb signals (O/T) could be used as an indicator for α-syn oligomerization.

While the AlphaLISA results are consistent with the TR-FRET results, AlphaLISA has a higher sensitivity and can detect lower concentration of α-syn (Fig. S3). The detection limit is about 50 ng/mL by TR-FRET using our antibody pair (Fig. S3A and S3B), whereas AlphaLISA can detect as low as 3.15 ng/mL (Fig. S3C and S3D). In addition, the oligomerization signals (O/T) clearly has a larger signal window when using AlphaLISA (Fig. 1H). Finally, AlphaLISA has been reported to be more tolerable to contaminants in clinical samples such as CSF, plasma, etc. (Bielefeld-Sevigny 2009), whereas TR-FRET failed to detect α-syn oligomers in CSF samples. Thus, AlphaLISA assay is likely superior than TR-FRET assay used for quantification of oligomerization.

We then measured the oligomeric α-syn, total α-syn AlphaLISA signal levels, and O/T ratio of human patient CSF from an independent cross-sectional cohort consisting of individuals diagnosed with PD, MSA, and age-matched control (Demographics, clinical features, and biomarkers’ level are listed in Table S1). There were no significant differences in oligomeric α-syn between PD, MSA, and control samples (*P* = 0.477) (Table S1, Fig. 2A). The total α-syn levels in CSF were significantly lower in MSA compared to control samples (*P* = 0.0184) (Fig. 2B). There is no significant difference between MSA and control CSF of O/T ratio. In contrast, the O/T ratio values of PD patients’ samples were significantly higher than the ones of control samples (*P* = 0.0164) (Fig. 2C). We also performed the correlation analysis of O/T with several factors including age, gender, RBC count, UPDRS-III score, and H&Y stage. The CSF O/T ratio has no correlation with age, gender, red blood cell (RBC) count, H&Y stage, and UPDRS-III score within the PD cohort (Fig. S4).

**Figure 2:**
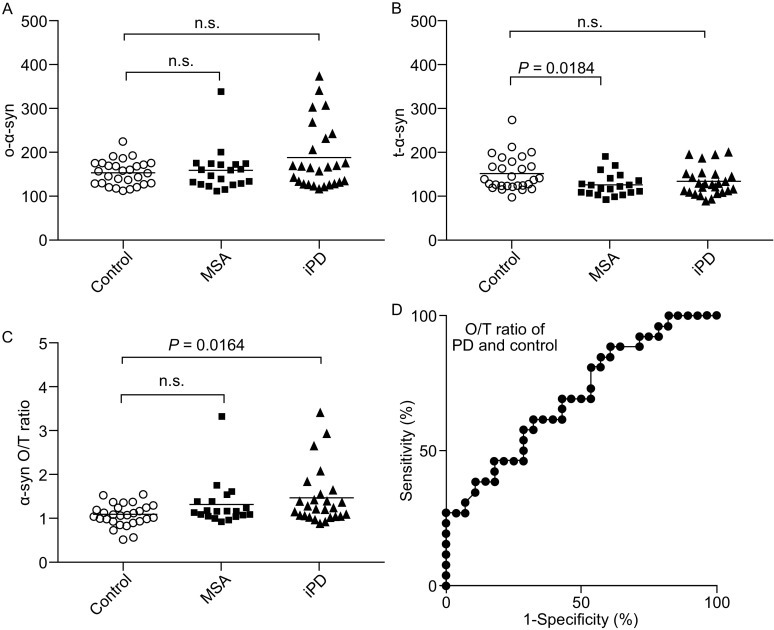
AlphaLISA assays detection of CSF α-syn in human patients. (A) Comparison of the oligomer α-syn between PD, MSA, and control patients. (B) Comparison of the total α-syn between PD, MSA, and control patients. (C) Comparison of the O/T ratio between PD, MSA, and control patients. (D) Receiver operating curves (ROC) for the levels of O/T in the patients tested CSF. All PD and control patients were concluded for ROC analysis, the area under curve (AUC) = 0.701. For Figure 2A–C, scatterplots representing individual sample values (means of 3 technical replicates). Each bar represents the mean value. PD = Parkinson’s disease, MSA = multiple system atrophy

We further tested the potential of using the O/T detected by AlphaLISA to provide auxiliary information for PD diagnosis. We plotted the receiver operating characteristic (ROC) curve using the data above obtained from PD and control patients, and the area under the ROC curve is 0.701, *P* = 0.012 (Fig. 2D). The cut-off levels of 1.155 for CSF O/T ratio yielded a sensitivity of 61.5% and a specificity of 67.9%.

In the present study, we established novel bead-based AlphaLISA assays to measure the relative levels of oligomeric and total α-syn in CSF samples, which is potentially useful for the diagnosis of patients with PD and MSA. Our data suggest that the O/T ratio of α-syn was significantly higher in patients with typical PD. The O/T ratio of α-syn may be served as an auxiliary biomarker for PD diagnosis (Fig. 2) and provides important information in the clinical practices. Using the O/T ratio of CSF α-syn, a sensitivity and specificity of 61.5% and 67.9% for the discrimination of patients with PD from controls could be reached based on our data in the current study.

## Electronic supplementary material

Below is the link to the electronic supplementary material.

Supplementary material 1 (PDF 292 kb)

Supplementary material 2 (PDF 1026 kb)
