# Supplementary material for: AlphaLISA detection of alpha-synuclein in the cerebrospinal fluid and its potential application in Parkinson’s disease diagnosis
Source: Protein Cell. 2017 May 29;8(9):696–700. doi: 10.1007/s13238-017-0424-4 (PMC5563284; doi:10.1007/s13238-017-0424-4)
Supplement: Supplementary file 1 — Supplementary material 1 (PDF 292 kb) [file 13238_2017_424_MOESM1_ESM.pdf]

Supplemental Information

Materials and Methods

Participants and CSF samples

CSF samples were obtained from 26 patients with idiopathic PD (ages 31-75, mean±SD=59.8±11.9), 20 patients with MSA (ages 45-72, mean±SD=57.4±6.8) and 28 age-matched neurologically normal control (ages 32-75, mean±SD=61.2±10.9). All PD patients fulfilled the criteria for idiopathic PD (iPD) according to UK Parkinson's Disease Society Brain Bank criteria (Hughes, Daniel et al. 1992). MSA patients diagnosed according to second consensus statement on the diagnosis of multiple system atrophy(Gilman, Wenning et al. 2008). Disease stage was determined by the Hoehn and Yahr stage (H&Y stage). The motor part (part III) of the UPDRS was used to assess the severity of disease by two independent trained interviewers during an "OFF" medication state, which was defined as being off anti-parkinsonism medications for at least 12 hours. All study subjects provided written informed consent to participate, which was approved by the University Ethics Committee （Huashan Hospital, Fudan University, Shanghai, China）.

A total of 12 mL of CSF was collected in a polypropylene tube between 8 AM and 10 AM under fasting condition through lumbar puncture. First 1–2 mL CSF was for basic CSF assessment, and last 10 mL CSF was for biobanking, collected in polypropylene tubes and gently mixed to avoid gradient effects. All samples were centrifuged within 30 min at 4 ℃ at 2,000 g for 10 min to remove cells and debris. The supernatant was collected and then stored at -80 ℃ until it defrosted for the TR-FRET and AlphaLISA. Repeat freeze/thaw cycles were avoided. CSF samples with more than 500 erythrocytes/mL were excluded from analysis(Teunissen, Petzold et al. 2009).

Preparation of α-syn wild type and mutant A53T

First, we cloned α-syn wild type and mutant A53T encoding fragment in prokaryotic pET expression vector. We also inserted a TEV protease cleavage site between N terminal His tag and the α-syn. The plasmid encoding α-syn wild type and mutant

A53T were verified and transformed into Escherichia coli BL21 (DE3) bacteria for expression. The cells were grown in Luria Bertani (LB) medium and induced by 0.5mM IPTG in 20℃ for 16 hrs. Then the cells were collected and loaded on a Ni-NTA column. After being eluted by imidazole, the N terminal His tag was removed by TEV protease treatment overnight in 4℃, the samples were buffer exchanged by ultracentrifuge and reloaded on a Ni-NTA column. The flow through fraction containing the His tag removed target proteins was further concentrated by ultracentrifuge. The molecular weight and purified α-syn/A53T proteins were confirmed by SDS-PAGE gel, NativePAGE gel and the concentration estimated using a BCA protein assay.

Purified α-syn protein and size exclusion chromatography samples were separated on SDS-polyacrylamide gels with 4-12% gradient and transferred onto PVDF membranes using the Trans-Blot Turbo transfer system (Bio-Rad, USA). The membranes were incubated with 5% milk in PBST buffer for 1h at room temperature to block unspecific binding and then incubated in primary antibody mouse-anti-α-syn diluted in blocking solution (1:500) overnight at 4℃. The next day the membranes were washed with PBST and incubated in appropriate peroxidase-labeled secondary antibody in blocking solution, and bands were visualized using the SuperSignal West-Pico Chemiluminescent Substrate (Pierce) and HyperFilm ECL (GE Healthcare).

To maintain target protein biological activity and ensure the protein status what we see is true, we also did NativePAGE and Western blot with 4-16% gradient gels. The Native PAGE™ Novex Bis-Tris gel system (Invitrogen) was used to perform electrophoresis under native conditions. Coomassie G250 was added to purified α-syn at a concentration of 0.25% as well as to the cathode buffer at concentrations of 0.02%, and 1-2ug of sample/lane was loaded. Protein was separated in NativePAGE™ Novex 4-16% Bis-Tris gels at 150V constant for 90 minutes 4℃ and transferred to PVDF at 70V for 60 minutes using the Mini Trans-Blot Cell (Bio-Rad).

Blots were treated with 8% acetic acid for 15 min, air-dried, then soaked in methanol to remove excess Coomassie Blue as well as to visualize the molecular mass markers. NativeMark™ Unstained Protein Standard is specifically designed for use with NativePAGE™ Novex® Bis-Tris Gels and consists of 8 protein bands that allow accurate molecular weight estimation in the range of ~20–1200 kDa. After washing PBS+0.1% Tween 20 (PBST), blots were processed for Western blot by blocking in 5% nonfat dry milk in PBST then incubated in primary antibody (the Life antibody) diluted in blocking solution (1:500) overnight at 4 ℃. Blots were incubated in peroxidase-labeled secondary antibody in blocking solution, and bands were visualized using the SuperSignal West-Pico Chemiluminescent Substrate (Pierce) and HyperFilm ECL (GE Healthcare).

Size Exclusion Chromatography

The concentrated His tag removed α-syn wild type and mutant A53T were further separated by size exclusive chromatography. The Superdex 200 10/300 GL column was equilibrated by PBS buffer, and then the samples were separated on the column with the speed of 0.4mL/min. The samples with different molecular weight was collected by fraction collector and verified by SDS-PAGE and Western blot.

Purified samples of α-syn in sterilized phosphate-buffered saline (PBS, pH7.4), in parafilm-sealed, 1.5mL Eppendorf tubes were incubated at 37 ℃ for several days in an Eppendorf Thermomixer with continuous mixing(1000rpm). Samples were collected at various time points and stored at -80 ℃ before loading onto the TR-FRET and AlphaLISA assay plates.

AlphaLISA assays

As shown in Fig.S1, an anti-analyte antibody which is biotinylated binds the Streptavidin Donor beads while another anti-analyte antibody is conjugated to AlphaLISA Acceptor beads. In the presence of the analyte, the beads come into close proximity. The excitation of the Donor beads provokes the release of singlet oxygen

molecules that triggers a cascade of energy transfer in the Acceptor beads, resulting in light emission.

The AlphaLISA assay was performed in 384-well microtiter plates (white opaque OptiPlate™ from Perkin Elmer) containing 5 μL of purified α-syn or cerebrospinal fluid

clinical samples, 5 μL of biotinylated Life or Nb monoclonal antibody, and 5 μL of Life antibody (syn211) conjugated to AlphaLISA acceptor beads, all in 1x AlphaLISA buffer. The reaction mixture was incubated at room temperature for 60min, then streptavidin donor beads (10 μL at 40μg/mL) were added and the plate incubated at room temperature in the dark for another 60min, after which it was read on an EnVision Reader (PerkinElmer) using the AlphaScreen protocol. Briefly, the AlphaScreen protocol used AlphaScreen label 384-well Packard OptiPlates and the AlphaScreen 570 emission filter, a flash/time ratio of 0.55, a measurement height of 1 mm, an excitation time of 0.18 s, and an emission time of 0.37 s. The initial optimization was performed in a final test volume of 25 μL per well and final concentrations of acceptor beads and donor beads of 10 μg/mL and 40 μg/mL, respectively. The biotinylated Life or Nb monoclonal antibody was titrated from 1 nM to 1 μM using the above final concentrations of donor and acceptor beads. The experiments were performed in triplicate.

Establishment of the oligomer and total α-syn specific assays

The antibodies we used for the TR-FRET and AlphaLISA assay to quantification of oligomeric and total α-syn were as follows: The Life antibody (Thermo Fisher Scientific, syn211, cat. no. 32-8100; it recognizes amino acid residues 121-125 of human α-syn), this mAb didn't react with recombinant β-synuclein or γ-synuclein on Western blots. Another antibody is based on the nanobody NbSyn2 (Nb antibody) developed by Erwin J. De Genst in 2010 which can recognize α-syn C-terminal nine amino acids (N-GYQDYEPEA-C) (De Genst, Guilliams et al. 2010). We cloned, expressed and purified the NbSyn2 protein by whole gene synthesize method and

purified by standard Ni-NTA and size exclusive chromatography protocol.

The antibodies were then biotinylated or conjugated to AlphaLISA acceptor beads as described below:

Biotinylation of antibodies:

Fresh 2mg/mL N-hydroxysuccinimido-ChromaLink-biotin (SoluLink Inc.) in PBS pH 7.4 was added to each antibody solution (BSA, glycerol and azide free) at 30:1 molar ratio. The volume was adjusted to 200 µL with PBS pH 7.4. After being incubated for 2 h at 23 ℃ in the dark, the antibody-biotin solutions were filtered through Zeba spin columns (Thermo Scientific, USA) at 1,500 g for 2 min to remove unbound biotin. Concentration of the antibody and biotinylation efficiency was measured using a Nano-Drop2000 instrument (Thermo Fisher Scientific, USA). Optical densities (OD) values at 280 and 354 nm were used to assess total protein and biotin concentrations, respectively. Biotinylated antibodies were stored at 4 ℃ at a concentration of 500nM in PBS with 0.05% NaN_3 and 0.1% Tween-20.

Acceptor-bead coupling of antibodies:

1mg of Europium Acceptor-beads (AlphaLISA Acceptor- beads, PerkinElmer,USA) were washed with PBS, centrifuged at 16,000 rcf for 15 min and resuspended in a 10:1 bead to antibody weight radio. The volume was adjusted to 200 µL, with 1 M Hepes pH 7.4, 10 % Tween-20 was added to reach a final concentration of 0.0625 % and freshly prepared 400mM $NaBH_3CN$ in H_2O to reach a final concentration of 20mM. Afterwards the antibody-bead solution was incubated for 24 h at 37 ℃. To block unreacted sites freshly made 65 mg/mL CMO in a 0.8 M NaOH was added at a final concentration of 3 mg/mL and incubated for 1 h at 37 ℃. The antibody-bead solution was centrifuged for 15 min at 16,000 g at 4 °C and washed twice with 200 µL, respectively, of 0.1 M Tris-HCl pH 8.0. Antibody-coupled Acceptor-beads were re-suspended at 5 mg/mL in storage buffer (200uL of PBS + 0.05 % Proclin-300), vortexed, briefly spin down and sonicated (10 short pulses of 1 second using a probe sonicator) and stored the conjugated Acceptor beads solution at 4 ℃ in an opaque

vial.

In all steps below, antibodies and beads were diluted in 1× assay buffer (10× AlphaLISA Immunoassay buffer: 250 mM HEPES, pH 7.4, 1 % Casein, 10 mg/mL Dextran-500, 5 % Triton X-100 and 0.5 % Proclin-300, PerkinElmer, USA) and samples were diluted using PBS pH 7.4. Each biotinylated antibody was combined with each Acceptor-bead coupled antibody.

TR-FRET assay for quantification of oligomer and total α-syn

The Life and Nb antibodies were chemically coupled to Lumi4-Tb (terbium cryptate, Cisbio) donor fluorophore or d2 acceptor fluorophore (Cisbio). Antibodies labeled with donor or acceptor fluorophores are denoted throughout this report by the suffixes –Tb or –d2, respectively. For assays, 384-well low volume polystyrene (Thermo Scientific) microtiter plates were loaded with 6μl analyte sample and subsequently mixed with 4 μL of antibody solution (50mM NaH_2PO4, 400mM KF, 0.1% BSA, and 0.05% Tween 20) which contained 0.023 μg/mL of –Tb antibody and 1.4 μg/mL of –d2 antibody. Plates were incubated at 4℃ overnight before measurement of time-resolved fluorescence at 620 and 665 nm on an Envision Multilabel reader (PerkinElmer Life Sciences).

Statistical analysis.

Statistical analyses were performed using GraphPad Prism (version 7.0) software and IBM SPSS software 23. To compare demographic and CSF data between groups, a nonparametric Kruskal-Wallis test was performed, followed by a Dunn’s multiple comparisons test was used. The Pearson χ^2 test was used for dichotomous variables. Correlation analyses were performed using the Spearman rank correlation. The accuracy of the diagnostic value of the biomarkers was assessed based on the area under the curve (AUC) of the receiver operating characteristic (ROC) curve. Cut-off values were calculated using sensitivity and specificity values that maximized Youden’s index (sensitivity + specificity − 1). All levels of significance were

two-tailed and set at $p < 0.05$.
