# Supplementary material for: AlphaLISA detection of alpha-synuclein in the cerebrospinal fluid and its potential application in Parkinson’s disease diagnosis
Source: Protein Cell. 2017 May 29;8(9):696–700. doi: 10.1007/s13238-017-0424-4 (PMC5563284; doi:10.1007/s13238-017-0424-4)
Supplement: Supplementary file 2 — Supplementary material 2 (PDF 1026 kb) [file 13238_2017_424_MOESM2_ESM.pdf]

Supplement of Figures and Table

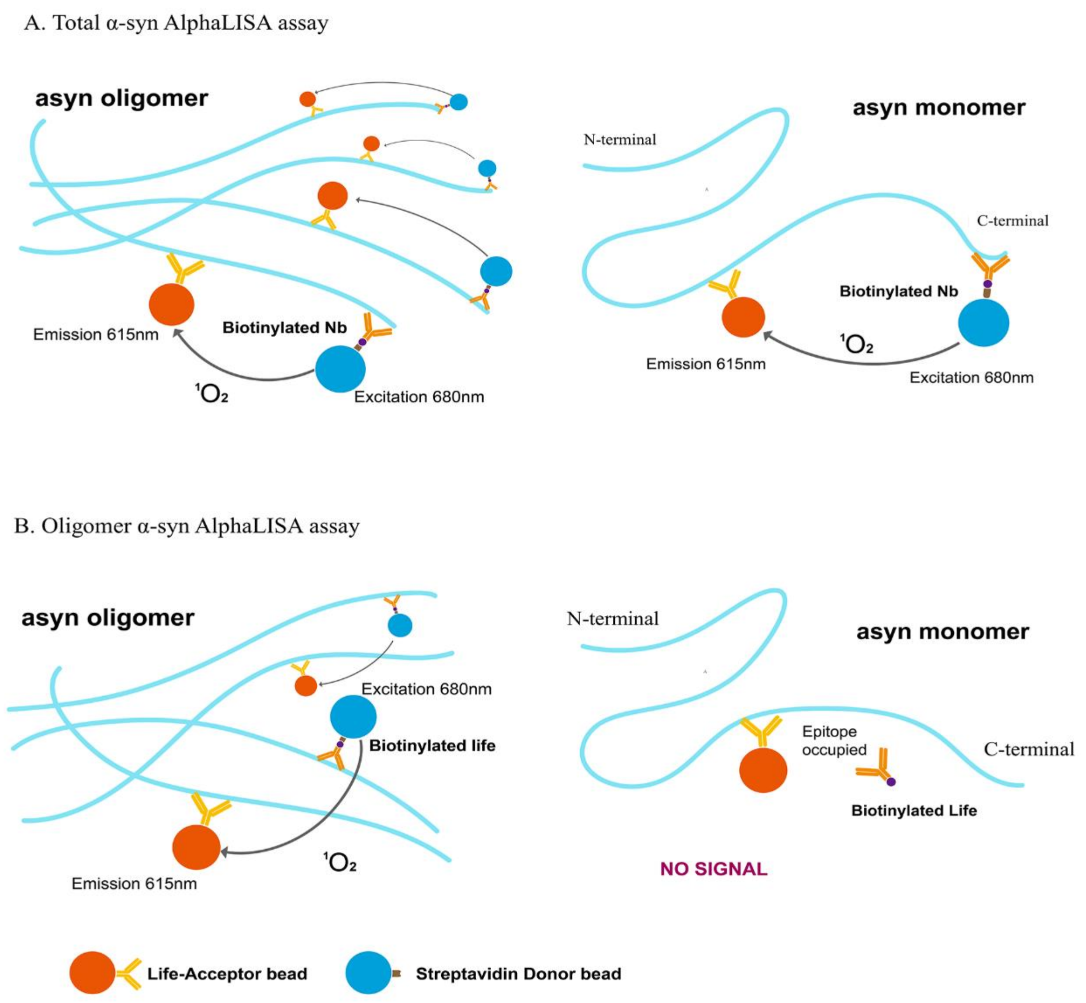

Figure S1 Schematic illustration of AlphaLISA assays for total and oligomeric α-syn models. (A) Total α-syn AlphaLISA assay: acceptor-beads (red) were conjugated to the Life antibody (targeting a.a. 121-125 of α-syn), whereas the Nb antibody was biotinylated and conjugated with the streptavidin-coated donor beads (blue). (B) Oligomer α-syn AlphaLISA assay, Life antibody coupled with Acceptor beads will not detect monomers due to occupancy of the same epitope by the biotinylated Life antibody. Only the oligomeric form of α-syn can provide multiple epitopes for binding of both the acceptor beads-conjugated and the donor beads-conjugated Life antibodies, so that AlphaLISA signals could be generated.

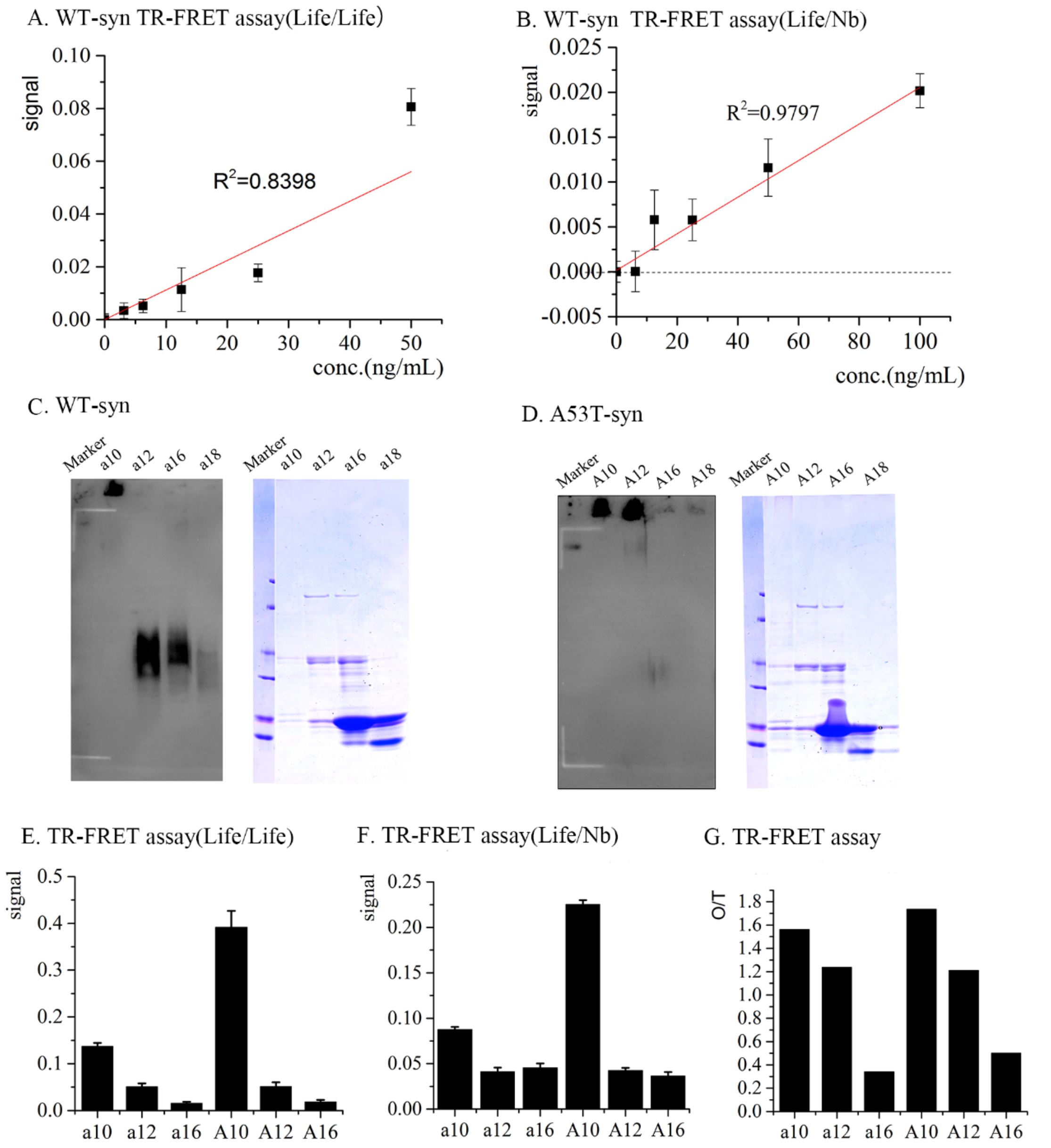

Figure S2 Development of AlphaLISA assays for detection of oligomeric and total α-syn. (A) TR-FRET assay of the purified protein in Fig.1A use the Life /Life antibody pair. The protein was tested at different concentrations indicated in the X-axis and the signals were fitted with Y=kX. R2 indicates the regression parameter. (B) Similar as A, but use the Life/Nb antibody pair. (C) Coomassie staining (blue) and western blot detection of indicated SEC-fractionated samples on NativePAGE, using the Life antibody. (D) Similar as C, but uses A53T protein. (E) The Life/Life antibody pair TR-FRET detection of the indicated α-syn protein samples from SEC at 1μg/mL on 384-well microtiter plates (n=3). The monomeric samples a12 & a16, A12 & A16 generated much smaller signals compared to oligomeric samples a10 and A10. (F) Similar as E, but use the Life/Nb antibody pair. All samples gave signals. (G) The ratio between Life/Life signals and the Life/Nb signals was calculated for the TR-FRET assay, and this ratio (O/T) separates oligomeric and monomeric samples. For E-G, plots indicate mean ±S. E, and n=3.

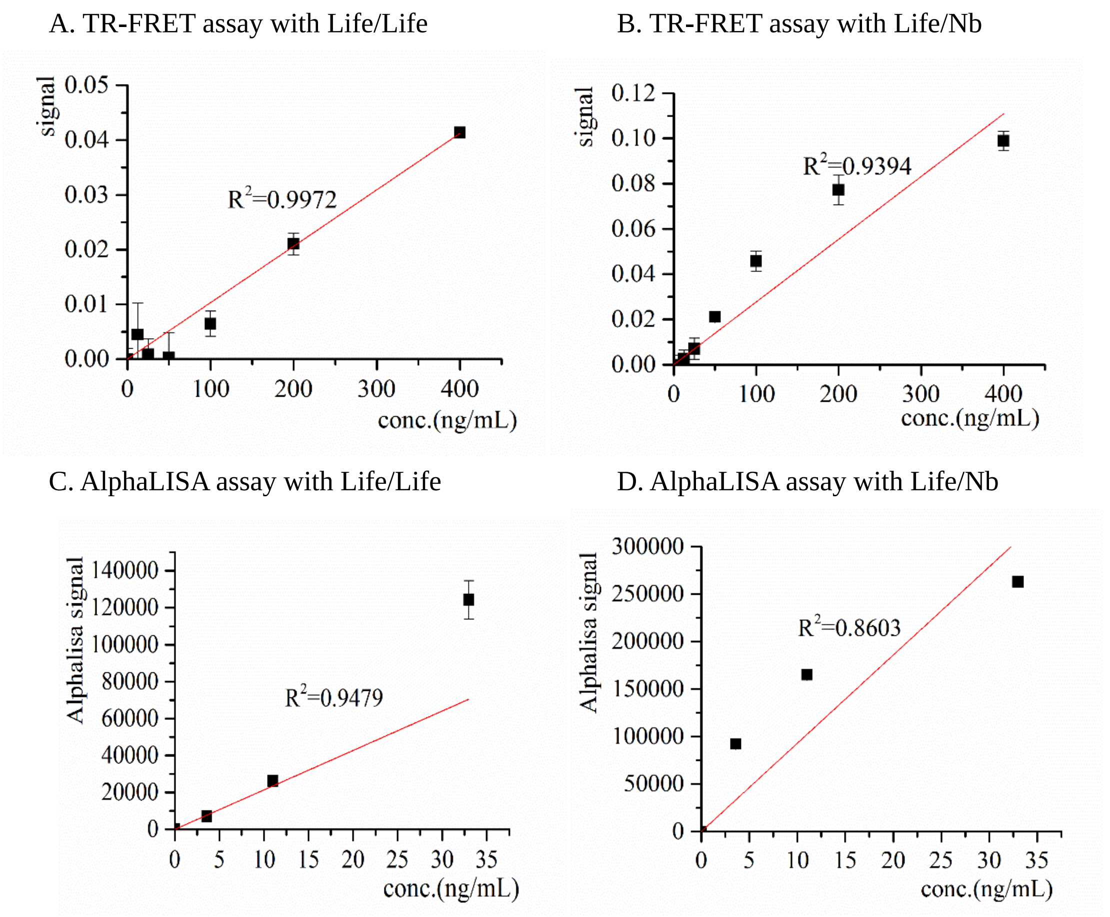

Figure S3 AlphaLISA has a higher sensitivity than TR-FRET and can detect lower concentration of α-syn. (A) TR-FRET assay of the purified protein in Fig.1A use the Life /Life antibody pair. The protein was tested at different concentrations indicated in the X-axis and the signals were fitted with Y=kX. R^2 indicates the regression parameter.

(B) Similar as A, but use the Life/Nb antibody pair. (C) Similar as A, but testing with the AlphaLISA assay using the Life/Life antibody pair. (D) Similar as C, but using the Life/Nb antibody pair. For A-D, plots indicate mean ±S. E, and n=3.

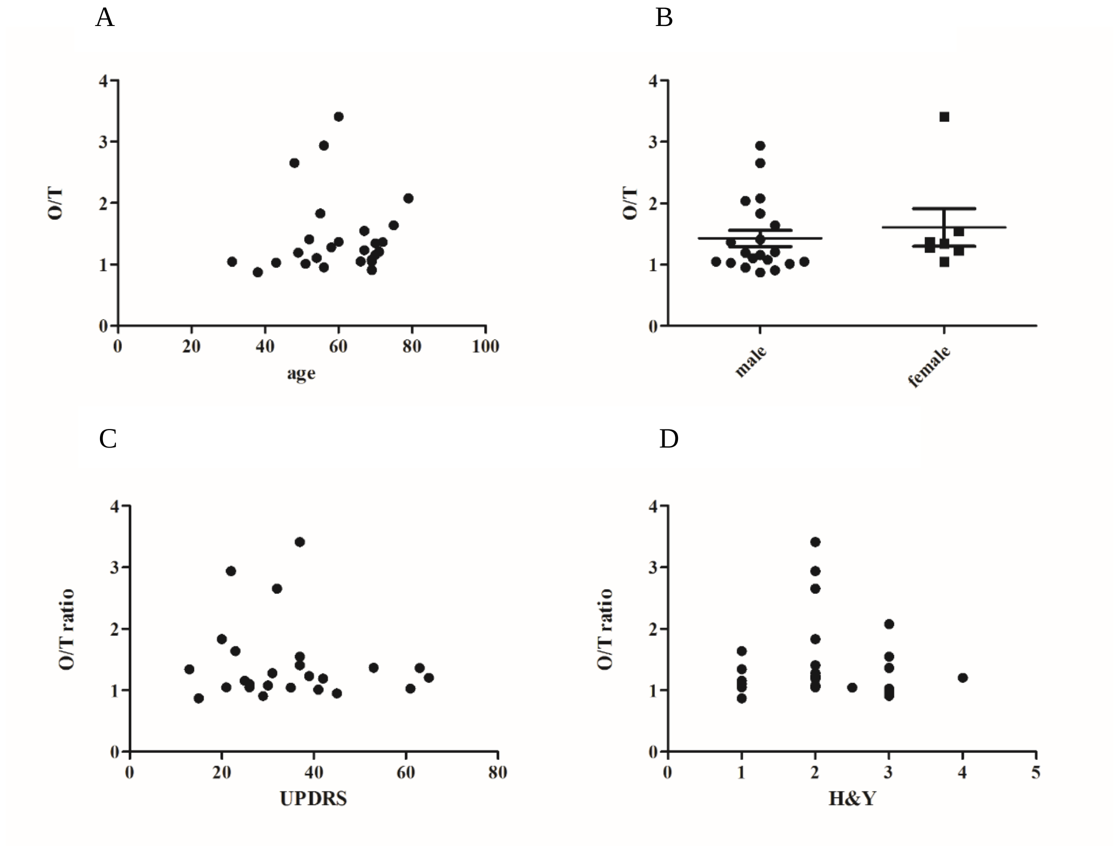

Figure S4 The correlation of the CSF O/T ratio with age, gender, H&Y value and UPDRS score within the PD cohort. (A) Age. (B) gender. (C) UPDRS. (D) H&Y. There has no correlation with these factors.

Table S1 Demographics, clinical features and biomarkers value

	PD（n=26）	MSA（n=20）	Control（n=28）	P value
Age(years)	59.8（11.9）	57.4（6.8）	61.2（10.9）	0.293
Gende(M/F)	19/7	11/9	19/9	0.426
Disease duration(month)	56.6（43.2）	29.0（22.0）	NA	
H&Y stage	2.2（0.8）	3.6（0.7）	NA	
UPDRS-III off score	34.7（14.2）	36.5（14.7）	NA	
Oligomer α-syn	187.0（74.9）	158.5（49.1）	153.3（28.2）	0.477
Total α-syn	132.8（31.5）	125.1（25.8）	150.5（39.3）	0.021*
O/T ratio	1.45（0.65）	1.31（0.52）	1.06（0.25）	0.026*

M=male, F=female, UPDRS = Unified Parkinson's Disease Rating Scale, H&Y= Hoehn and Yahr stage; Data are shown as mean (SD)
